# Comparative insights into the mechanism of ultrasonic-assisted chlorogenic acid grafting and carboxymethylation on the emulsifying properties of palm kernel expeller globulin

**DOI:** 10.3389/fnut.2026.1804894

**Published:** 2026-03-20

**Authors:** Luchen Wang, Yong Yang, Biao Ma, Yashu Wu, Yan Li, Qinping Yang, Jiayu Liu

**Affiliations:** 1 Food Science College of Shanxi Normal University, Taiyuan, China; 2 Shanxi Province Cancer Hospital, Taiyuan, China; 3 Shanxi Hospital Affiliated to Cancer Hospital, Chinese Academy of Medical Sciences, Taiyuan, China; 4 Cancer Hospital Affiliated to Shanxi Medical University, Taiyuan, China

**Keywords:** carboxymethylation, chlorogenic acid grafting, emulsion characterization, mechanism insights, palm kernel expeller globulin, ultrasonication

## Abstract

**Background:**

Palm kernel expeller globulin (PKEG) is an abundant and underutilized protein with promising potential as a natural emulsifier, provided its emulsifying properties can be enhanced.

**Methods:**

This study assessed the effects and mechanisms of two ultrasonic-assisted emulsification enhancement techniques on PKEG: chlorogenic acid grafting and carboxymethylation on the emulsifying properties of PKEG.

**Results:**

Compared to ultrasonic-assisted chlorogenic acid grafting, ultrasonication-assisted carboxymethylation was more effective at enhancing the emulsifying activity (from 57.06–109.42 m^2^/g) and emulsion stability (64.72–90.00%) of PKEG. This was achieved by decreasing its molecular mass, increasing the random coil content (25.9–52.4%), solubility (59.14–86.85 g/100 g), and interface adsorption capacity (58.23–245.61 μg/mL), reducing droplet size (1.24–0.69 μm) and the loss tangent of the emulsion; and augmenting the absolute zeta potential (−33.07 to −76.54 mV), centrifugal stability (26.59–74.14%), and viscosity. Ultrasonication-assisted chlorogenic acid grafting also increased the random coil and *β*-sheet contents, hydrophobicity (from 276.49–351.38), and interface adsorption capacity of PKEG; decreased the droplet size and loss tangent; and enhanced zeta potential. These effects contributed to the enhanced emulsifying activity (57.06–82.54 m^2^/g) and emulsion stability (64.72–89.96%) of PKEG, albeit to a lesser extent than found with carboxymethylation. Moreover, ultrasonication-assisted chlorogenic acid grafting decreased the pH sensitivity of PKEG’s solubility and emulsifying activity.

**Conclusion:**

These findings indicate that PKEG modified by ultrasonication-assisted chlorogenic acid grafting or carboxymethylation has potential for development as novel emulsifiers.

## 1 Introduction

Palm (*Elaeis guineensis* Jacq) kernel expeller is a byproduct of palm oil processing with an annual yield of approximately 430 million tons (1). Although palm kernel expeller is rich in protein (23 g/100 g), its applications in the food industry remain limited (2). Currently, a small portion of palm kernel expeller is used as feed and fertilizer, while the majority is discarded, resulting in substantial resource waste and environmental pollution (3). Globulin accounts for 61.75 g/100 g of palm kernel expeller proteins (4), which comprises antihypertensive, antioxidant, antibacterial, and iron-fortification peptides (5, 6). Moreover, palm kernel expeller globulin (PKEG) exhibits considerable emulsifying activity (64.18 m^2^/g) and emulsion stability (79.46%) without potential toxicity (7); however, its emulsifying properties remain inferior to those of other sources, such as soy protein (8). Thus, PKEG can offer a safe and efficient emulsifier if its emulsifying properties can be effectively improved. However, to our knowledge, there have been few studies devoted to understanding and enhancing the emulsifying properties of PKEG. Such insights could facilitate the utilization of other plant-derived compounds (such as from palm kernel and *Ruta graveolens* L.) for their diverse phytochemical and pharmacological benefits in functional food design (9).

Emulsifiers play a crucial role in food nutrition and human health. Emulsifiers are crucial for the processing, transportation, digestion, and absorption of nutrients such as lipids, fat-soluble vitamins, and cholesterol (10). Moreover, emulsifiers facilitate the formation of emulsions between immiscible liquids by adsorbing at the water–oil interface and forming an interfacial film (11). Compared to chemically synthesized emulsifiers, natural protein emulsifiers offer advantages in safety, nutritional value, environmental sustainability, and availability (12). However, plant protein-based emulsifiers have some drawbacks, including relatively lower efficiency, poor solubility or hydrophobicity, and susceptibility to environmental factors (13). To address these limitations, various chemical, biological, and physical approaches have been employed to enhance the emulsifying properties of plant proteins (8, 14). Chlorogenic acid is a natural and safe polyphenol found in various plant species (15), which can improve the hydrophobicity and interfacial properties of plant proteins upon binding (16, 17). Specifically, the phenolic hydroxyl group and hydroxyl group in the carboxyl group of chlorogenic acid can form C–N and C–O–C covalent bonds with the amino and carboxyl groups of proteins, respectively, thereby promoting the proteins’ hydrophobicity and interface sorption properties, resulting in better antioxidant and emulsion properties (18). Furthermore, carboxymethylation has been used to enhance the functionality of proteins by introducing hydrophilic carboxymethyl groups into the protein structure (19). Ultrasonication generates mechanical vibration and cavitation effects to expose more functional groups, thereby increasing the emulsifying properties of proteins (20). Other modification methods have also proven to be effective in improving protein interface properties, such as gallic-binding, high-pressure processing, and glycosylation; however, carboxymethylation and chlorogenic acid grafting have emerged as the most safe and effective approaches for improving the emulsifying properties of proteins (11, 19, 21, 22). Compared to traditional methods, ultrasonication-assisted carboxymethylation or chlorogenic acid-grafting offers greater cost-effectiveness and is a more environmentally friendly approach. However, information on the effects and mechanisms of combining these modification methods on the emulsifying properties of proteins remains scarce.

To address this gap, we evaluated the effects of ultrasonication-assisted chlorogenic acid grafting or carboxymethylation on the emulsifying properties of PKEG and explored the underlying mechanisms. This study can therefore help to advance the utilization of PKEG while establishing a foundation for the rational design of high-value, plant-based emulsifiers from underutilized agro-industrial residues.

## 2 Materials and methods

### 2.1 Materials

The palm kernel expeller was obtained from Bigzhipo Oil Palm Processing Company (Haikou, China). Monochloroacetic acid, Nile Blue A sulphate, 8-anilino-1-naphthalenesulfonic acid (ANS), disodium hydrogen phosphate, chlorogenic acid (purity> 99.5%), and other reagents were of analytical grade and purchased from Soliabo Reagents Co. Ltd. (Tianjin, China). Glycine, Coomassie Blue R-250, acrylamide, sodium dodecyl sulfate (SDS), and tetramethylethylenediamine were of electrophoretic purity and purchased from Yijun Co. (Beijing, China). Alcalase (1.0 × 10^4^ U/mg) and protein markers were purchased from Sinopharm Biochemical Institute (Nanjing, China).

### 2.2 Extraction of PKEG

Globulin was extracted from the palm kernel expeller using 0.2 mol/L of NaCl (1:25 g/mL) according to the protocol reported by Zheng et al. (23). The extraction rate (g/100 g) was defined as the amount of PKEG obtained per weight of palm kernel expeller.

### 2.3 Ultrasonication of PKEG

According to a modified method of Liu et al. (24), PKEG (5 g) in 250 mL of phosphate buffer (0.1 mol∙L^−1^, pH 7.5) was vortexed at 1,200 rpm using a HulaMixer™ Mixer (Thermo Fisher Scientific Co., Waltham, MA, USA) for 12 s. The dispersion was then subjected to ultrasonic treatment at 80 kHz, 45 ± 1 °C, and 400 W (power density of 8.45 W/mL) using a LanJ-L45 Ultrasonic cleaner (inner tank size of 450 × 300 × 300 mm; Jialang Ultrasonic Electrical Appliance Co. Ltd., Guangzhou, China) for 45 min. After lyophilization with an AFD150 lyophilizer (Shengke Vacuum Dry Instrument Factory, Nanjing, China), the PKEG modified by ultrasonication (PKEG-U) was obtained.

### 2.4 Carboxymethylation of the ultrasonically treated PKEG

Following the modified protocol reported by Xu et al. (1), 120 mL of the ultrasonically treated PKEG solution was mixed with 1.51 mol/L of NaOH (15 mL, dissolved in 85% ethanol). The mixture was then shaken at 205 rpm and 53 °C using an ASYH-2 oscillator (Beifa Shaking Instrument Co. Ltd., Zhenjiang, China) for 1 h. Subsequently, 1 mL of chloroacetic acid (1.17 mol/L) was added. The solution was stirred at 35 °C for 0.5 h, and continuously stirred at 55 °C for 2.5 h. The pH value of the solution was reduced to 7.0. After 48 h of dialysis against deionized water (dH_2_O) using a dialysis membrane (cutoff of 4 kDa) at 4 °C, the dialysate was lyophilized with the AFD150 lyophilizer to obtain the ultrasonic-assisted carboxymethylation-treated PKEG (PKEG-UC). The carboxymethyl degree was measured according to the protocol of Wang et al. (25).

### 2.5 Carboxymethylation of PKEG

Carboxymethylation of PKEG was performed using the same protocols as described in section 2.4. The degree of substitution of the obtained carboxymethylated palm kernel expeller globulin (PKEG-C) was measured according to the protocol of Wang et al. (25). Chlorogenic acid grafting of the ultrasonically treated PKEG.

Following the modified procedure of Ye et al. (17), 120 mL of the ultrasonically treated PKEG solution was mixed with 0.15 g of ascorbic acid and 1 g of chlorogenic acid. The solution was stirred at 160 rpm and 25 °C for 110 min. Subsequently, 1 mol/L of H_2_O_2_ (2 mL) was added, and the reaction was continued at 115 rpm and 35 °C under a nitrogen flow for 16 h. After centrifugation at 5,200 × *g* for 0.5 h, the supernatant was collected and mixed with five volumes of acetone, which was then centrifuged again at 5,200 × *g* for 25 min. The precipitate was collected and dialyzed against dH_2_O using a dialysis membrane (cutoff of 4 kDa) at 4 °C for 36 h. The retentate was lyophilized with the AFD150 lyophilizer to obtain the PKEG modified via ultrasound-assisted chlorogenic acid grafting (PKEG-UCA). The yield rate (g/100 g) was expressed as the obtained PKEG-UCA per 100 g of dry PKEG. The degree of substitution was determined using the Folin–Ciocalteu method (26).

### 2.6 Chlorogenic acid grafting of PKEG

Chlorogenic acid grafting of PKEG was performed using the same protocol as described in section 2.6. The substitution degree of the obtained PKEG chlorogenic acid conjugation (PKEG-CA) was determined based on the Folin–Ciocalteu method (26).

### 2.7 Chemical constitution analysis

The moisture, fat, ash, and protein content of PKEG, PKEG-U, PKEG-CA, PKEG-UCA, PKEG-C, and PKEG-UC were determined according to standard methods of the AOAC.924.05, AOAC.920.39, AOAC.92.05, and AOAC.955.04, respectively (27). Phenolic content was determined according to the Folin–Ciocalteu method (26).

### 2.8 Structural characteristics

#### 2.8.1 Sodium dodecyl sulphate polyacrylamide gel electrophoresis (SDS-PAGE)

A Mini-G4 Vertical electrophoresis system (Bio-Rad, Shanghai, China) was used for SDS-PAGE analysis according to standard protocols from Laemmli (28). The separating gel, stacking gel, and sample concentrations were 12.5, 5%, and 1 mg/mL, respectively. The gel was stained with Coomassie Blue-R250 (10 mg/mL) for 12 h. The molecular weight was analyzed using a BOXF3 Gel imaging system (Synene, Tokyo, Japan) with rabbit phosphorylase *b* (97.4 kDa), bovine serum albumin (66.2 kDa), rabbit actin (43.0 kDa), carbonic anhydrase (31.0 kDa), trypsin inhibitor (20.1 kDa), and egg white lysozyme (14.4 kDa) as mass markers.

#### 2.8.2 Fluorescence spectra

The PKEG samples (250 μg each) were dissolved in 1 mL of phosphate buffer (pH 7.0, 10 mmol/L) (29). The endogenous fluorescence spectrum of PKEG was scanned using a Y-F100 fluorescence spectrophotometer (Iphisong Technology Co., Ltd., Tianjin, China) with a wavelength scanning range of 200–500 nm and an excitation wavelength of 290 nm. The excitation and emission slits were both set to 5 nm.

#### 2.8.3 Secondary structure analysis

The circular dichroism analysis of PKEG, PKEG-U, PKEG-CA, PKEG-UCA, PKEG-C, and PKEG-UC was conducted using a JASCO-1500 circular dichroism spectrometer (JASCO Corporation, Tokyo, Japan) with a wavelength range of 190–260 nm (30). The concentration of the PKEGs and scanning rate were 100 μg/mL and 100 nm/min, respectively. The obtained data were analyzed using CDNN 2.1-Simple Spectra software.

#### 2.8.4 Fourier-transformed infrared (FT-IR) spectroscopy

PKEGs (1 mg) and dry KBr (150 mg) were mixed thoroughly and pressed to form a thin slice (1–2 mm), which was loaded onto a Nolay-20 FT-IR spectrometer (Nolayda Technology Co. Ltd., Qingdao, China). Scanning was performed over a wavenumber range of 4,000–400 cm^−1^ with a resolution of 4 cm^−1^ (31).

### 2.9 Solubility under various pH

The PKEG dispersions (2 g/100 mL, dispersed in dH_2_O) were each adjusted to pH 2, 4, 6, 8, and 10, respectively (20). These dispersions were stirred at 25 ± 1 °C and 160 rpm in the ASYH-2 oscillator for 0.5 h. The dispersions were centrifuged at 3,547 × *g* at 25 °C, and the supernatants were pooled. The protein concentration was measured according to the Bradford method (32), and the solubility was calculated using Equation 1:

$$\;\;\;\;\text{Solubility}\;(g/100\;g)=(C_{S}\times V_{S})/W_{M}\times 100\;\;$$ (1)

Where *C_S_* and *V_S_* are the protein concentration and volume of the supernatant, respectively; *W_M_* is the weight of PKEG.

### 2.10 Surface hydrophobicity

Following the method of De la Cruz-Torres et al. (33), 20 μL of ANS (8 mmol/L, pH 7.8) was added to 2 mL of each PKEG solution prepared at different concentrations (10–600 μg/mL). Fluorescence measurement was conducted using an FL6500 spectrophotometer (PerkinElmer Inc., MA, USA) with excitation, divergence, and gap wavelengths of 390 nm, 470 nm, and 5 nm, respectively. Hydrophobicity was taken as the initial section of the fluorescence profile as a function of PKEG concentration.

### 2.11 Emulsion characterization and mechanistic evaluation

#### 2.11.1 Preparation and stability of the PKEG-based emulsions

To prepare the emulsions, 45 mL of PKEG solution (4.5 g/100 mL) and 15 mL of soybean oil were homogenized at 20,000 rpm with an HSD-I40 homogenizer (Lihu Homogenizing Instrument Co., Wuxi, China) according to the protocol of Pearce and Kinsella (34). After 120 s, the emulsion (40 μL) was mixed with 1 mg/mL of SDS solution (2,600 μL). The absorbance was measured at 500 nm (*A_0_*) and again 10 min later (*A_t_*). The emulsifying activity and emulsion stability were calculated using Equations 2, 3, respectively:

$$\begin{array}{l} \text{Emulsifiability}\;(m^{2}/g)=(2.303\times 2\times A_{0}\times f)/\\ (C\times \varphi \times 10,000)\;\;\; \end{array}$$ (2)

$$\text{Emulsion stability}\;(\%)=A_{t}/A_{0}\times 100$$ (3)

Where 2 is a constant accounting for the interfacial area being twice the turbidity; 2.303 refers to the value of ln(10); and *f*, *C*, and *φ* represent the dilution factor (50), PKEG content (g/mL), and proportion of soybean oil, respectively.

#### 2.11.2 Cryogenic scanning electron microscopy

Based on the method of Abou-Elsoud et al. (35), 1 mL of each of the fresh PKEG-based emulsions was mixed with 20 μL of Nile Blue A sulphate (1 mg/L) and 20 μL of 9-(diethylamino)-benzo-*α*-phenoxazin-5(5H)-one (1 mg/L, dissolved in isopropyl alcohol). After reaction for 0.5 h in the dark, 12 μL of the mixture was pipetted on a glass slide and observed using an LSM880-Airyscan Zeiss cryogenic laser confocal scanning electron microscope (Carl Zeiss AG, Baden-Wurttemberg, Germany) with excitation wavelengths of 633 and 488 nm, respectively. The profiles were photographed at a magnification of 40 ×.

#### 2.11.3 Interface adsorption ability

Following the protocol of Ravindran et al. (36), the PKEG-based emulsions (2 mL) were centrifuged at 8,460 × *g* for 20 min, and the resulting aqueous phase in the upper layer was pipetted out and pooled. This centrifugation process was repeated three times. The pooled upper layer was used to determine protein concentration according to the Bradford method (32). Interface adsorption ability was calculated using Equation 4:

$$\text{Interface adsorption ability}=(C_{0}-C_{f})\;$$ (4)

where *C_0_* and *C_f_* are the initial and final protein concentrations in the aqueous phase, respectively.

#### 2.11.4 Particle size and zeta potential

Following the method of Nobakht-Nia, Niakousari, Eskandari, Golmakani, & Hosseini (37), the volume- and area-weighted average particle size (D_3,2_) of the PKEGs-based emulsion was analyzed using a Malvern Panco Nanoparticle Size and Zeta Potential Analyzer (Zetasizer Pro, Malvern Panalytical Co., Ltd., Malvern, England). Moreover, the emulsions stabilized by PKEGs were diluted 100-fold with dH_2_O. The dilution was gently poured into a U-shaped cuvette, and the zeta potential was determined using the Zetasizer Pro-ZETA Potential Analyzer at 25 °C.

#### 2.11.5 Centrifugal stability

The centrifugal stability of the PKEG-based emulsions was determined according to the method of Xing et al. (29). The initial absorbance (*A_0_*) of each emulsion (10 mL) was determined at 500 nm. The PKEGs-based emulsions were then centrifuged at 5,000 × *g* for 20 min, and 10 μL of the bottom phase was withdrawn and mixed with 3 mL of dH_2_O. The absorbance of the resulting solution (*A_t_*) was recorded at 500 nm, and centrifugal stability was calculated using Equation 5:

$$\text{Centrifugal stability}=(A_{0}-A_{t})/A_{0}\times 100$$ (5)

#### 2.11.6 Rheological properties

A DHR-3 Rheometer (Baosheng Industrial Development Co., Ltd., Shanghai, China) equipped with a PP50 parallel plate and a P-PTD 200 temperature controller was used to measure the rheological properties of the PKEG-UC emulsion. Samples were placed on the parallel plate and equilibrated at 25 °C for 5 min to eliminate residual loading stresses (29). Viscosity measurement was carried out over a shear rate range of 0.1–300 s^−1^. Subsequently, dynamic frequency scanning was conducted under the following conditions: linear rate of 0.1–100 rad/s, gap distance of 1 mm, strain amplitude of 0.1%, oscillation frequency of 0.1–10 Hz, and stress of 0.1–1,000 Pa. Storage modulus (*G*’), loss modulus (*G*”), and loss tangent (tan *δ* = *G*”/*G*’) were recorded.

#### 2.11.7 Three-phase contact angle

Following the method of Nobakht-Nia et al. (37), a CIA150 Contact Angle Measuring Instrument (SunXian Haihou Instrument Co., LTD., Wuxi, China) was used to determine the three-phase contact angles of emulsions stabilized using PKEG, PKEG-UCA, and PKEG-UC. Briefly, approximately 25 μL of the emulsion was slowly dripped onto the sample platform, forming a film with a thickness of approximately 1 mm and a diameter of 10 mm. Subsequently, the film surface was sprinkled with approximately 10 μL of ultrapure water using a syringe needle, and the surface was photographed after 6 s. The contact angles were calculated using the Laplace–Young equation.

### 2.12 Statistical analysis

All experiments were conducted in at least triplicate. Statistical significance was assessed using one-way analysis of variance followed by Tukey’s post-hoc test (IBM SPSS Statistics v14, IBM Corp., Armonk, NY, USA). *p* < 0.05 indicated a statistically significant difference among groups.

## 3 Results and discussion

### 3.1 Chemical composition and modification degree of PKEG, PKEG-UC, and PKEG-UCA

**Figure 1:**
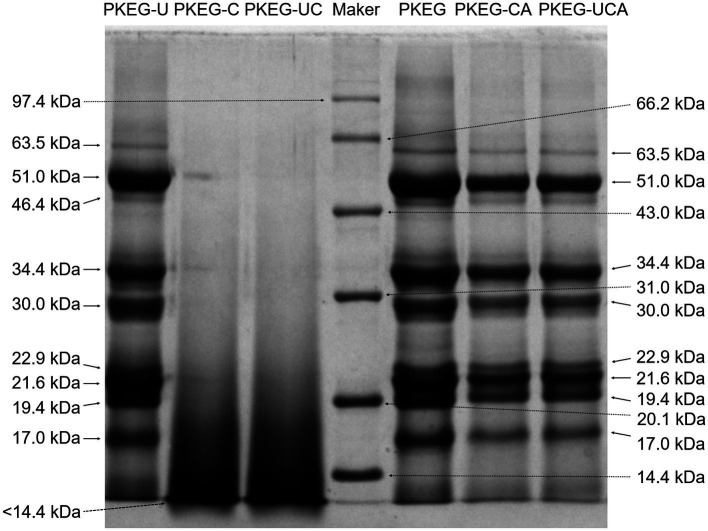
SDS-polyacrylamide gel electrophoresis diagrams of PKEG, PKEG-U, PKEG-C, PKEG-UC, PKEG-CA, and PKEG-UCA were performed using 12.5% separating gel and 5% stacking gel. PKEG; PKEG-U, PKEG-modified via ultrasonication; PKEG-CA, PKEG-modified via chlorogenic acid-grafting; PKEG-UC, PKEG-modified via ultrasonication-assisted carboxymethylation; and PKEG-UCA, PKEG-modified via ultrasonication-assisted chlorogenic acid-grafting. Protein markers included rabbit phosphorylase *b* (97.4 kDa), bovine serum albumin (66.2 kDa), rabbit actin (43.0 kDa), carbonic anhydrase (31.0 kDa), trypsin inhibitor (20.1 kDa), and egg white lysozyme (14.4 kDa).

The extraction rate of PKEG was 12.77 ± 0.39 g/100 g of palm kernel expeller, indicating that 87.29% globulin was extracted. A prior study obtained a similar result of 14.16 g/100 g (7). Zhang et al. (38) found that ultrasonication improved the extraction rate of *Ginkgo biloba* polysaccharides; thus, the effects of ultrasonication on the extraction of PKEG should be studied further. Furthermore, since ultrasound outcomes strongly depend on power density, probe vs. bath configuration, vessel geometry, sample depth, and temperature control, the specific effect of ultrasonication on PKEG properties should be investigated further. The yield rates of PKEG-UC and PKEG-UCA were 79.47 ± 3.78 and 82.00 ± 4.19 g/100 g PKEG, respectively. As shown in Table 1, the substitution degrees of PKEG-UC and PKEG-UCA were 6.05 and 9.29%, respectively, confirming that carboxymethyl groups and chlorogenic acid were successfully grafted onto the PKEG, respectively. Furthermore, the degree of substitution of PKEG-UCA was higher than that of PKEG-CA (4.79% ± 0.26%), while the degree of substitution of PKEG-UC exceeded that of PKEG-C (3.12% ± 0.17%), indicating that ultrasonication enhanced both carboxymethylation and chlorogenic acid grafting efficiency. The cavitation and thermal effects of ultrasonication can induce structural unfolding of PKEG (39), thereby increasing accessibility of carboxymethyl and chlorogenic acid groups to bind to the reactive sites on PKEG. No statistically significant differences (*p* > 0.05) were observed in moisture, ash, or protein contents among PKEG, PKEG-U, PKEG-CA, PKEG-UCA, PKEG-C, and PKEG-UC. Conversely, ultrasonication alone decreased the fat content of PKEG; moreover, chlorogenic acid grafting, carboxymethylation, and ultrasonication-assisted chlorogenic acid grafting significantly reduced the fat and fiber contents (*p* < 0.05). The cavitation effect of ultrasonication, along with alkali and heating treatments used during carboxymethylation, caused the degradation of polypeptide chains in PKEG to break its spatial structure (Figure 1), resulting in loss of fat and fiber (35). Additionally, the higher extractable total phenol content of PKEG-CA and PKEG-UCA relative to PKEG (Table 1) supported successful chlorogenic acid conjugation (17). Notably, PKEG-UCA exhibited a higher phenol content than PKEG-CA, further confirming the facilitating role of ultrasonication in chlorogenic acid grafting. Carboxymethylation and ultrasonication-assisted carboxymethylation increased the total phenol content of PKEG, probably ascribed to the introduction of carboxymethyl groups. Xu et al. (1) demonstrated that carboxymethylation increased the phenolic acid content of millet fiber.

**Table 1:** Effects of ultrasonication-assisted carboxymethylation or chlorogenic acid-grafting on the proximate composition and molecular weight of palm kernel expeller globulin.

Proximate composition	Palm kernel expeller	PKEG	PKEG-U	PKEG-C	PKEG-UC	PKEG-CA	PKEG-UCA
Substitution degree (%) ^a^	–	Control	–	3.12% ± 0.17%	6.25 ± 0.18c	4.79% ± 0.26%	9.29 ± 0.37b
Molecular weight distribution (kDa)	–	17.0–63.5	17.0–63.5	< 14.4	< 14.4	17.0–63.5	17.0–63.5
Moisture (g∙100 g^−1^)	8.70 ± 1.52b	4.67 ± 0.62c	5.33 ± 0.09c	5.05 ± 0.42c	4.50 ± 1.31c	6.09 ± 0.44c	5.00 ± 0.79c
Fat (g∙100 g^−1^)	5.91 ± 0.19b	2.79 ± 0.65c	1.58 ± 0.32d	1.46 ± 0.09d	1.39 ± 0.78d	1.08 ± 0.03d	1.56 ± 0.21d
Ash (g∙100 g^−1^)	5.42 ± 0.67b	1.82 ± 0.39c	2.00 ± 0.11c	2.09 ± 0.32c	2.75 ± 0.32bc	2.79 ± 0.15bc	2.11 ± 0.45c
Protein (g∙100 g^−1^)	23.69 ± 1.44c	90.60 ± 2.40b	91.21 ± 3.76b	92.53 ± 3.33b	91.20 ± 5.60b	92.00 ± 2.59b	93.87 ± 5.67b
Soluble carbohydrate (g∙100 g^−1^)	11.03 ± 0.17b	1.09 ± 0.04c	0.97 ± 0.11c	1.14 ± 0.03c	1.02 ± 0.06c	1.12 ± 0.09c	0.91 ± 0.09c
Crude fiber (g∙100 g^−1^)	39.88 ± 4.49b	1.49 ± 0.13c	1.14 ± 0.13d	0.94 ± 0.05d	0.72 ± 0.07d	1.05 ± 0.22d	0.73 ± 0.11d
Extractable total phenols (mg∙g^−1^)	1.87 ± 0.09d	2.01 ± 0.06e	2.47 ± 0.24de	4.05 ± 0.28d	3.73 ± 0.03d	9.64 ± 0.37c	11.48 ± 0.16b

a Substitution degree of carboxymethyl groups and ferulic acid on PKEG. PKEG-1, palm kernel expeller globulin; PKEG-U, palm kernel expeller globulin modified via ultrasonication; PKEG-UC, palm kernel expeller globulin modified via ultrasonication-assisted carboxymethylation; PKEG-CA, palm kernel expeller globulin modified via chlorogenic acid-grafting; PKEG-UCA, palm kernel expeller globulin modified via ultrasonication-assisted chlorogenic acid-grafting. “–,” not measured. Different lowercase letters (b–e) in the same row indicate a significant difference (*p* < 0.05).

### 3.2 Structural analysis

#### 3.2.1 SDS-PAGE

As depicted in Figure 1, nine subunits with molecular masses of 63.5–17.0 kDa were identified in PKEG. Among them, subunits with molecular weights of 51.0, 34.4, and 21.6 kDa accounted for 29.71, 19.98, and 18.86 g/100 g, respectively (Table 2), which is consistent with the findings of Zheng et al. (7). PKEG-U retained the same nine subunits as native PKEG (63.5–17.0 kDa). Except for the subunits with a mass of 34.4 and <14.4 kDa, no significant differences (*p* > 0.05) were detected in the content of the remaining eight subunits between PKEG and PKEG-U (Table 2), indicating that the ultrasonic treatment (80 kHz and 400 W, 45 min) did not induce substantial subunit degradation. By contrast, the contents of subunits with masses of 51.0, 34.4, and 21.6 kDa were only 1.43, 0.72, and 0.5%, respectively. PKEG-UC contained only one subunit (< 14.4 kDa), revealing that the alkali and heating treatments (1.15 mol/L of NaOH, 53 °C) applied during carboxymethylation induced degradation of PKEG and lowered its molecular mass. PKEG-CA and PKEG-UCA contained the same nine subunits found in PKEG (63.5–17.0 kDa), but the contents of subunits with molecular masses of 63.5, 46.4, and 51.0 kDa were dramatically decreased (Table 2). Furthermore, no statistically significant differences in subunit distribution were observed between PKEG-CA and PKEG-UCA, indicating that ultrasonication-assisted chlorogenic acid grafting, not ultrasonication alone, caused the degradation of PKEG, but to a lesser degree than that induced by carboxymethylation, which is attributed to the relatively mild reaction conditions (18). A decrease in molecular mass can enhance the emulsifying properties of PKEG, as smaller peptides/proteins can migrate faster to the interface and form a film to stabilize the emulsion droplets (21).

**Table 2:** Molecular weight distribution and content of subunits of palm kernel expeller globulin and the palm kernel expeller globulins modified via ultrasonication (PKEG-U), carboxymethylation (PKEG-C), chlorogenic acid-grafting (PKEG-CA), and ultrasonication-assisted chlorogenic acid-grafting (PKEG-UCA) or carboxymethylation (PKEG-UC), respectively.

Subunit number	Molecular weight (kDa)	Content (g/100 g)					
		PKEG	PKEG-U	PKEG-C	PKEG-UC	PKEG-CA	PKEG-UCA
1	63.5	3.61 ± 0.19a	4.05 ± 0.17a	0.00 ± 0.00c	0.00 ± 0.00c	0.55 ± 0.04b	0.49 ± 0.03b
2	51.0	29.71 ± 1.67a	25.99 ± 2.43a	1.43 ± 0.07c	0.00 ± 0.00d	20.67 ± 2.33b	20.49 ± 1.41b
3	46.4	1.78 ± 0.05a	0.54 ± 0.11b	0.00 ± 0.00c	0.00 ± 0.00c	0.36 ± 0.05c	0.27 ± 0.033c
4	34.4	19.98 ± 1.26b	17.65 ± 2.04b	0.72 ± 0.09c	0.00 ± 0.00d	21.51 ± 0.74a	22.41 ± 1.39a
5	30.0	9.77 ± 0.42a	8.68 ± 0.33a	0.00 ± 0.00b	0.00 ± 0.00b	13.15 ± 0.39a	12.00 ± 0.42a
6	22.9	9.67 ± 0.34a	8.76 ± 0.25a	0.00 ± 0.00b	0.00 ± 0.00b	7.04 ± 0.37a	8.71 ± 0.45a
7	21.6	18.86 ± 1.29b	19.45 ± 0.18b	0.50 ± 0.03c	0.00 ± 0.00d	21.77 ± 1.15a	21.46 ± 0.76a
8	19.4	9.67 ± 0.34a	8.13 ± 1.01a	0.00 ± 0.00b	0.00 ± 0.00b	7.93 ± 0.38a	8.79 ± 0.37a
9	17.0	6.62 ± 0.72a	5.74 ± 0.36a	0.00 ± 0.00b	0.00 ± 0.00b	7.02 ± 0.47a	6.14 ± 0.13a
10	< 14.4	0.00 ± 0.00c	1.01 ± 0.00b	97.35 ± 0.55a	100.00 ± 0.00a	0.00 ± 0.00c	0.00 ± 0.00c

Different lowercase letters (a–c) in the same row indicate a significant difference (*p* < 0.05).

#### 3.2.2 Endogenous fluorescence spectra

**Figure 2:**
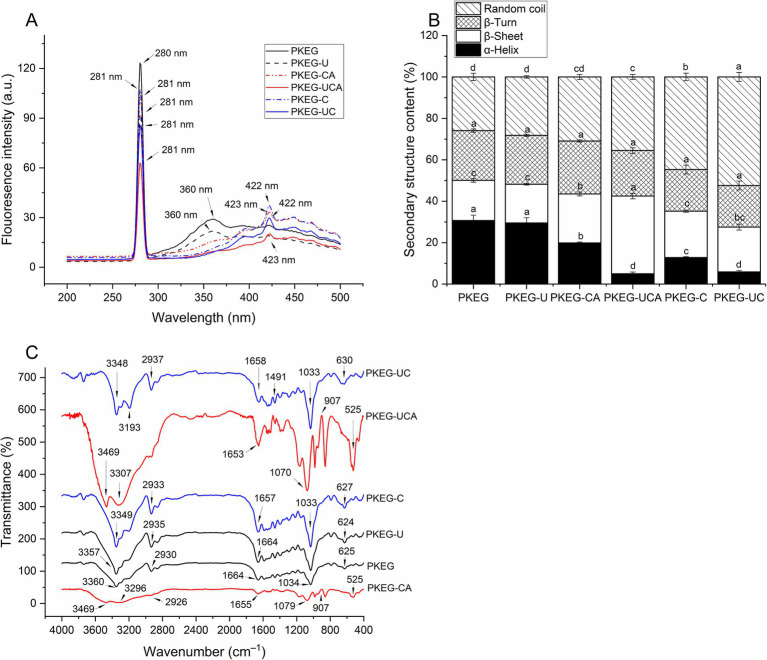
Influences of ultrasonication, chlorogenic acid grafting, carboxymethylation, and ultrasonication-assisted chlorogenic acid-grafting or carboxymethylation on the structural properties of PKEG. **(A)** Endogenous fluorescence spectra; **(B)** Secondary structure composition; and **(C)** Fourier-transform infrared spectroscopy of PKEG, PKEG-U, PKEG-C, PKEG-UC, PKEG-CA, and PKEG-UCA. Different lowercase letters (a–d) above on bars mean significant difference (*p* < 0.05).

As shown in Figure 2A, notable differences were evident among the fluorescence spectra of PKEG, PKEG-U, PKEG-CA, PKEG-UCA, PKEG-C, and PKEG-UC. The sharp peak at 280 nm in the spectrum of PKEG [corresponding to the fluorescence adsorption of Ser and Trp; Tang et al. (22)] slightly shifted to 281 nm in the fluorescence spectra of PKEG-U, PKEG-CA, PKEG-UCA, PKEG-C, and PKEG-UC. Another peak of PKEG at 360 nm (indicative of the fluorescence adsorption of Ser) moved to 422 nm, 423 nm, 422 nm, and 423 nm in the spectra of PKEG-C, PKEG-CA, PKEG-UCA, and PKEG-UC, respectively, indicating that ultrasonication, chlorogenic acid-grafting, carboxymethylation, and ultrasonication-assisted chlorogenic acid-grafting or carboxymethylation caused the arrangement of hydrophobic amino acids in PKEG (30). Ultrasonication, alkaline, and heating treatments during carboxymethylation caused the degradation of PKEG’s subunits (Figure 1). It altered the spatial structure, leading to the arrangement of hydrophobic groups and different fluorescence spectra (18).

#### 3.2.3 CD analysis

As shown in Figure 2B, ultrasonication did not alter the secondary structure of PKEG. However, chlorogenic acid grafting, carboxymethylation, and ultrasonication-assisted chlorogenic acid-grafting or carboxymethylation induced measurable changes in secondary structural composition. The *α*-helix (a rigid structure) content of PKEG substantially decreased, whereas the random coil (a loose structure) content increased. Ultrasonication, alkaline, and heating treatments applied during carboxymethylation can interrupt hydrogen bonds and disrupt the ordered structure (α-helix) of PKEG, increasing the random coil content. Furthermore, the random coil content of PKEG-UCA and PKEG-CA was lower than that of PKEG-UC and PKEG-C, which may be attributed to the relatively mild reaction conditions during chlorogenic acid-grafting. Compared with PKEG, PKEG-UCA and PKEG-CA exhibited a higher content of *β*-sheet (*p* < 0.05). A β-sheet is a relatively more extended structure than an α-helix, which is largely maintained by hydrogen bonds (40). After chlorogenic acid-grafting, the introduced phenolic hydroxyl groups promoted the formation of intramolecular and intermolecular hydrogen bonds, thereby facilitating the reorganization of β-sheets (13). These findings demonstrated that chlorogenic acid grafting, carboxymethylation, and ultrasonication-assisted chlorogenic acid grafting or carboxymethylation loosened the structure of PKEG. Additionally, PKEG-UC showed a higher random coil content than both PKEG-U and PKEG-C (*p* < 0.05), suggesting a synergistic effect of ultrasonication and carboxymethylation in increasing the structural flexibility of PKEG. A more extended structure was conducive to protein adsorption at the water–oil interface, thereby forming stable emulsions (41).

#### 3.2.4 FT-IR analysis

As shown in Figure 2C, distinct variations were observed across the FT-IR spectra of PKEG, PKEG-U, PKEG-CA, PKEG-UCA, PKEG-C, and PKEG-UC. The peak at 3360 cm^−1^ in the FT-IR spectrum of PKEG shifted to 3,348, 3,357, 3,469, 3,349, and 3,469 cm^−1^ in the spectra of PKEG-UC, PKEG-U, PKEG-CA, PKEG-C, and PKEG-UCA, respectively. Concurrently, new peaks appeared at 3193, 3296, and 3,307 cm^−1^ in the spectra of PKEG-UC, PKEG-CA, and PKEG-UCA. Moreover, the peak at 1662 cm^−1^ for PKEG (representative of C=O bond vibration) moved to 1,658, 1,657, 1,655, and 1,653 cm^−1^ in the spectra of PKEG-UC, PKEG-C, PKEG-CA, and PKEG-UCA, respectively, indicating that carboxymethylation, chlorogenic acid grafting, and ultrasonication-assisted chlorogenic acid grafting or carboxymethylation altered the N–H and –OH bonds in the amide band I of PKEG (30). Furthermore, a noticeable shift (from 2,930 to 2,933 and 2,937 cm^−1^) and a new peak at 1491 cm^−1^ in the spectra of PKEG-C and PKEG-UC were detected, indicative of the asymmetric stretching of C–H in methyl and carboxyl groups, respectively, reflecting the introduction of carboxymethyl groups (25). Compared to the spectrum of PKEG, there was a blue shift from 1,033 to 1,070 and 1,079 cm^−1^, a new peak at 907 cm^−1^, and a red shift from 625 to 525 cm^−1^ in the spectra of PKEG-CA and PKEG-UCA, corresponding to C–O, C–C bends, and benzene ring vibration, respectively (10, 18), confirming the successful grafting of chlorogenic acid on PKEG. Furthermore, the peaks at 2933, 1657, and 630 cm^−1^ in the spectrum of PKEG-C shifted to 2,937, 1,658, and 627 cm^−1^ in the spectrum of PKEG-UC, respectively; similarly, the peaks at 3296, 1655, and 1709 cm^−1^ in the spectrum of PKEG-CA shifted to 3,193, 1,653, and 1,070 cm^−1^ in the spectrum of PKEG-UCA, indicating that ultrasonication synergistically amplified the structural effects of both carboxymethylation and chlorogenic acid grafting. However, determining the more specific binding sites for PKEG–chlorogenic acid conjugates requires further investigation.

### 3.3 Solubility with varying pH

**Figure 3:**
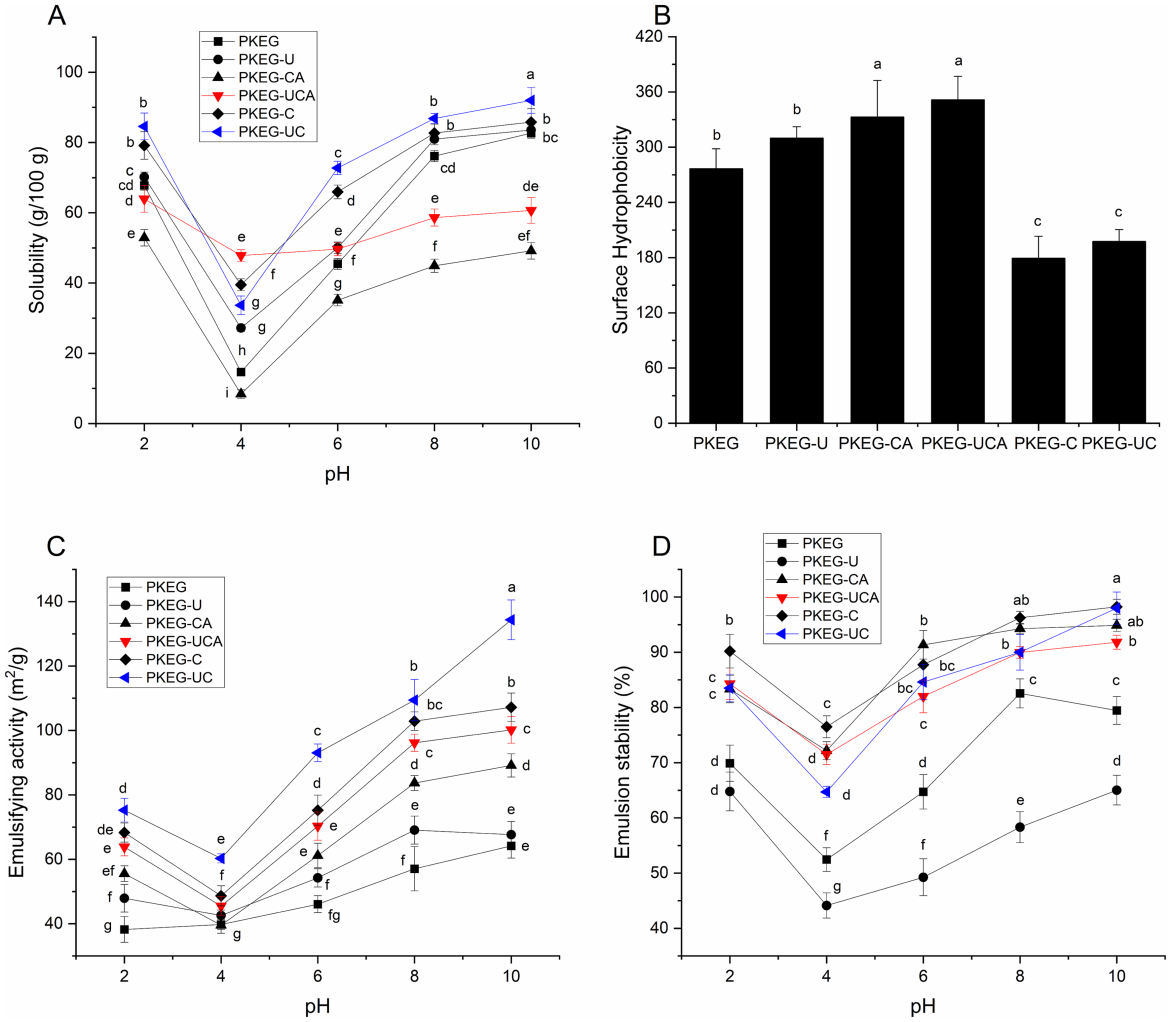
Solubility **(A)**, surface hydrophobicity **(B)**, emulsifying activity **(C)**, and emulsion stability **(D)** of PKEG, PKEG-U, PKEG-C, PKEG-UC, PKEG-CA, and PKEG-UCA at different pH values. Different lowercase letters (a–g) on the bars or data points indicate statistically significant differences (*p* < 0.05).

As shown in Figure 3A, PKEG, PKEG-U, PKEG-CA, PKEG-C, and PKEG-UC exhibited the lowest solubility at pH 4.0, which is the reported isoelectric point of PKEG (4). At the isoelectric point, the net charge of proteins is zero, and the electrostatic repulsion between proteins is the lowest, leading to aggregation and sediment formation (31). These PKEGs displayed increasing solubility as the pH increased from 6 to 10. Such an alkali pH-shift can expand a protein’s structure, exposing more polar groups and thereby increasing solubility (31).

Moreover, the solubility of PKEG-C and PKEG-UC was higher than that of PKEG at pH 2–10 (*p* < 0.05). Following carboxymethylation and ultrasonication-assisted carboxymethylation, the molecular mass of PKEG decreased (Figure 1), and its structure became looser (Figure 2B), thereby exposing more hydrophilic groups and increasing the affinity of PKEG to water. Additionally, the introduced carboxymethyl groups are hydrophilic (25), which contributed to the higher solubility of PKEG-UC. Furthermore, PKEG-UC exhibited higher solubility than PKEG-C and PKEG-U (*p* < 0.05), indicating that ultrasonication-assisted carboxymethylation is more effective in improving the solubility than either treatment alone. A similar result was obtained by Zhang et al. (14). Alternatively, PKEG-CA exhibited the lowest solubility at pH 2.0–10.0 among these PKEGs, and PKEG-UCA showed lower solubility than that of PKEG at pH 8.0–10.0, which can be ascribed to the poor hydrophilicity of chlorogenic acid (17). Notably, the solubility of PKEG-UCA was higher than that of PKEG-CA at pH 2.0–10.0, confirming that ultrasonication enhanced the solubility of PKEG. The lowest solubility of PKEG-UCA appeared at pH 4.0–6.0, indicating that ultrasonication-assisted chlorogenic acid grafting altered the isoelectric point of PKEG. Similarly, previous reports have shown that the solubility of soybean protein and Tartary buckwheat protein decreased following polyphenol binding (15, 39).

### 3.4 Surface hydrophobicity analysis

The surface hydrophobicity of PKEG was enhanced by chlorogenic acid grafting and ultrasonication-assisted chlorogenic acid grafting (Figure 3B), which is ascribed to the higher hydrophobicity of chlorogenic acids (31). Moreover, as described above, chlorogenic acid grafting and ultrasonication-assisted chlorogenic acid grafting altered the structure of PKEG (Figures 1, 2A–C), inducing the rearrangement of hydrophobic groups in PKEG and thereby enhancing its hydrophobicity (12). Ultrasonication increased the surface hydrophobicity of PKEG; however, this change was not statistically significant (*p* > 0.05). The cavitation effect of ultrasonic treatment can induce protein unfolding, thereby exposing buried hydrophobic groups (24). By contrast, PKEG-UC and PKEG-C had lower hydrophobicity than PKEG (*p* < 0.05), corresponding to their higher solubility (Figure 3A). Similarly, Wang et al. (25) found that carboxymethylation decreased the hydrophobicity of starch.

### 3.5 Emulsifying characteristics

Carboxymethylation, chlorogenic acid grafting, and ultrasonication-assisted chlorogenic acid grafting or carboxymethylation resulted in noticeable improvements in both the emulsifying activity and emulsion stability of PKEG at pH 2–10 (Figures 3C,D). Chlorogenic acid grafting and ultrasonication-assisted chlorogenic acid grafting enhanced the surface hydrophobicity of PKEG, whereas carboxymethylation and ultrasonication-assisted carboxymethylation enhanced solubility (Figures 3A,B), increasing its affinity for water and oils and improving its interface adsorbing capacity. Moreover, the phenolic hydroxyl groups of PKEG-UCA and PKEG-CA facilitate the reduction of the free energy and form a resilient interfacial film on the surface of oil droplets (10). Both PKEG-UC and PKEG-C exhibited higher emulsifying activity at pH 6–10 and better emulsion stability at pH 10 than PKEG-UCA and PKEG-CA, predominantly ascribed to their low molecular mass (<14.4 kDa, Figure 1), higher solubility, and increased random coil content (Figures 2B, 3A). A decrease in mass along with increases in flexibility and hydrophilicity can help proteins disperse quickly at an oil–water interface and form a stable film surrounding droplets (14, 35). Furthermore, PKEG-UC exhibited higher emulsifying activity than PKEG-C at pH 2–10, confirming that ultrasonication enhanced the efficiency of carboxymethylation in improving the emulsifying activity of PKEG. Conversely, the emulsion stability of PKEG-U was the poorest among all samples, as ultrasonication can cause aggregation of droplets on the oil–water interface (20).

PKEG, PKEG-U, PKEG-CA, PKEG-UCA, PKEG-C, and PKEG-UC exhibited the lowest emulsifying activity and emulsion stability at pH 4.0, and all these emulsions showed increasing emulsifying activity and emulsion stability as the pH increased from 6 to 10. This trend matched the observed change in solubility with pH (Figure 3A), confirming that solubility profoundly affects the emulsifying properties of proteins (41). Proteins have the lowest net charge at their isoelectric point and exhibit the poorest ability to prevent aggregation of the droplets (13). Increasing the pH promoted the dissociation of PKEG, thereby increasing the net charge and repulsive forces between droplets and enhancing the emulsion stability (36). Furthermore, compared with those of PKEG, the solubility and emulsifying property profiles of PKEG-UCA against pH were relatively gentler (Figures 3A–D), indicating that ultrasonication-assisted chlorogenic acid grafting reduced the sensitivity of PKEG to pH, given that the introduced chlorogenic acids altered the dissociation state of the protein (18). Additionally, the emulsifiability of PKEG-UC and PKEG-UCA (134.35 and 100.18 m^2^/g, respectively) was higher than that of soy protein (63.21 m^2^/g). The emulsion stability (91.81 and 97.89%, respectively) was nearly equal to that of polysorbate-80 (a commercial emulsifier) and better than that of soy protein (76.58%) (8), indicating their potential as efficient emulsifiers and emulsion stabilizers.

The data presented in Figures 1–3 demonstrated that ultrasonication, carboxymethylation, chlorogenic acid grafting, and ultrasonication-assisted chlorogenic acid grafting or carboxymethylation improved the emulsifying properties of PKEG by expanding its structure, lowering molecular weight, and increasing solubility or hydrophobicity. Ultrasonication enhanced the improvement effects of carboxymethylation and chlorogenic acid grafting on the emulsifying properties of PKEG. This study evaluates the effects of ultrasonication-assisted chlorogenic acid grafting or carboxymethylation on the emulsifying properties of PKEG and elucidates the associated underlying mechanisms. Accordingly, the fundamental mechanisms of ultrasonication, carboxymethylation, and chlorogenic acid grafting are beyond the scope of this investigation.

### 3.6 Mechanistic insights

#### 3.6.1 Cryogenic scanning electron microscopy of PKEG-based emulsions

**Figure 4:**
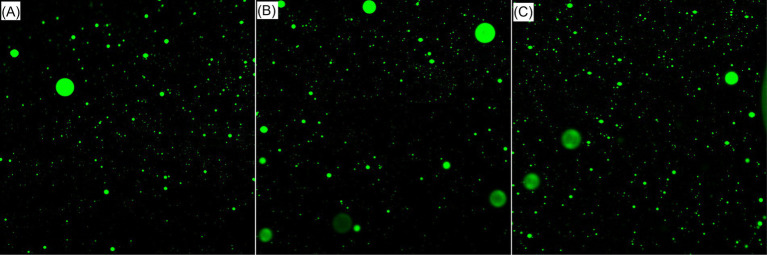
Cryogenic laser confocal scanning electron microscopy of the emulsions based on PKEG **(A)**, PKEG-UCA **(B)**, and PKEG-UC **(C)** at a magnification of 40 × with a scale bar of 50 μm. Emulsions were stained with Nile red and Nile Blue A Sulfate.

**Figure 5:**
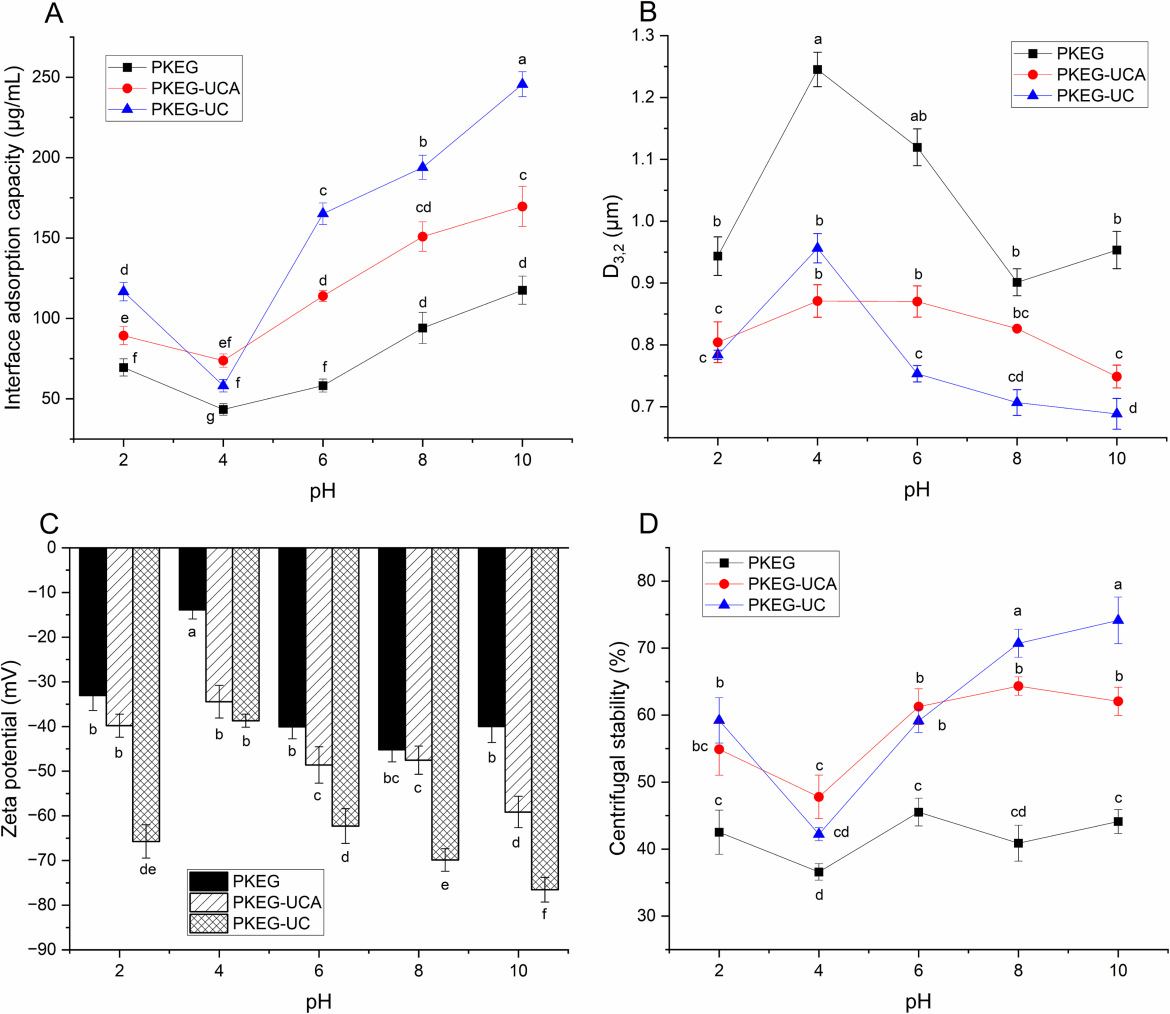
Mechanism insights of the emulsions stabilized using PKEG, PKEG-UCA, and PKEG-UC at various pH values. **(A)** Interfacial adsorption capacity of PKEG, PKEG-UCA, and PKEG-UC; **(B)** particle size of droplets, **(C)** zeta potential, and **(D)** centrifugal stability of the emulsions stabilized by PKEG, PKEG-UCA, and PKEG-UC, respectively. Different lowercase letters (a–g) on the bars or data points denote statistically significant differences (*p* < 0.05).

As illustrated in Figure 4, there were numerous droplets detected in the PKEG-based emulsion, consistent with the considerable emulsifying activity (64.18 m^2^/g) and emulsion stability of PKEG (79.46%, Figures 3C,D). More spherical emulsion droplets with smaller particle sizes were evident in the PKEG-UCA or PKEG-UC-based emulsions, corresponding to the better emulsifying activity and emulsion stability of PKEG-UC and PKEG-UCA. Moreover, the increased number of red spherical droplets (Figures 4B,C) suggested that ultrasonication-assisted chlorogenic acid-grafting or carboxymethylation improved the ability of PKEG to form a resilient emulsion film (11). Furthermore, the PKEG-UC-based emulsion had the greatest number of droplets (Figure 4C), which is ascribed to its highest emulsifying activity, emulsion stability, and interface adsorption capacity (Figures 3C,D, 5A). Similar findings were reported by Liu et al. (19) and Kim et al. (10).

#### 3.6.2 Interface adsorption ability of PKEGs

The change trend of the interface adsorption capacity of PKEG, PKEG-UCA, and PKEG-UC with a change in pH from 2 to 10 (Figure 5A) matched the change in emulsifying activity with varying pH (Figure 3C), confirming that interface adsorption capacity is crucial in contributing to emulsifying properties (14). PKEG-UC and PKEG-UCA exhibited higher interface adsorption capacity than that of PKEG at pH 2–10 (*p* < 0.05), which is attributed to their higher solubility and hydrophobicity (Figures 3A,B). Moreover, the higher random coil content (Figure 2B) was helpful for PKEG-UC and PKEG-UCA to adsorb on the water–oil interface and form an emulsion film (10). After ultrasonication-assisted carboxymethylation, the molecular mass decreased, and the spatial structure of PKEG changed (Figures 1, 2B), which exposes more hydrophilic or hydrophobic groups, thereby leading to a higher interfacial adsorption capacity. Qin et al. (21) also found that a decrease in molecular mass increased the interface adsorption ability of sunflower protein.

Furthermore, PKEG, PKEG-UCA, and PKEG-UC exhibited the lowest interface adsorption capacity at pH 4.0, which increased with pH from 6 to 10. At pH 4.0, the PKEGs had the lowest net charge and poor affinity for water and oils (30), exhibiting low interface adsorption capacity. An alkali pH-shift promoted protein dissociation, increased the surface charge, and induced structural rearrangements of PKEGs [Chen et al. (43); Ma et al. (11)], thereby exposing more polar or hydrophobic groups and enhancing the interface adsorption capacity. Additionally, PKEG-UC and PKEG-UCA exhibited higher interface adsorption capacities (161.61 and 295.61 μg/mL) than reported for soy protein [95.33 μg/mL; Ballabio et al. (42)].

#### 3.6.3 Particle size distribution

Larger emulsion droplets tend to aggregate and settle (37). The emulsions stabilized by PKEG, PKEG-UCA, and PKEG-UC exhibited a similar particle size curve against pH, with the largest D_3,2_ detected at pH 4.0 (Figure 5B). The charge in the double layer covering the droplets was lowest at pH 4.0 (near the isoelectric point); consequently, repulsive forces between the droplets decreased, leading to emulsion aggregation and larger-sized droplets (29). The D_3,2_ of the PKEG-based emulsions decreased as pH increased from 6 to 10 because protein dissociation was enhanced at higher pH and the net charge increased, thickening the dielectric double layer and increasing repulsion force (11), thereby decreasing particle size.

Moreover, the PKEG-UC and PKEG-UCA-based emulsions exhibited smaller D_3,2_ values than that of the PKEG-based emulsion (*p* < 0.05). Following ultrasonication-assisted chlorogenic acid-grafting or carboxymethylation, the structure of PKEG became looser (Figure 2B) and its interface adsorption capacity increased (Figure 5A), thereby increasing the double-layer thickness and the zeta potential (Figure 5C), resulting in a smaller D_3,2_ (42). At pH 6–10, the PKEG-UC-based emulsion had the smallest D_3,2_ value, corresponding to its superior emulsion stability (Figure 3D). Kim et al. (10) found that grafting *Tasmannia lanceolata* phenolic acids increased the hydrophobicity of pea protein, enhanced its affinity for oils and ability to adsorb at the water–oil interface, thereby increasing the zeta potential and repulsive forces between droplets, resulting in smaller droplets. This same mechanism was found in this study.

#### 3.6.4 Zeta potential

A decrease in zeta potential reduces the repulsive force between droplets and causes emulsion instability (13). The PKEG-UC-based emulsion showed a higher absolute zeta potential than that of PKEG at pH 2–10 (*p* < 0.05) (Figure 5C), contributing to its better emulsion stability (Figure 3D). After ultrasonication-assisted carboxymethylation, the hydrophilicity and interfacial adsorption capacity of PKEG were increased (Figures 3A, 5A), thereby increasing the electrostatic charge quantity on the slipping plane of the double layer (29) and ultimately leading to higher zeta potential. Furthermore, the zeta potential of OPKEG-UCA was higher than that of PKEG at pH 4, 6, and 10, contributing to its higher emulsion stability (Figure 3D). Collectively, the higher hydrophobicity (Figure 3B) and interface adsorption activity of PKEG-UCA (Figure 5A), along with the smaller droplet size (Figure 5B), resulted in an increase in charge quantity in the double layer and a higher zeta potential (8).

Moreover, the zeta potential of the PKEGs-based emulsions was lowest at pH 4.0, increasing with pH from 6 to 10. At pH 4.0, the net charge of PKEGs and the repulsive force between the droplets were the lowest, thereby decreasing the zeta potential (20). However, the pH shift (6–10) promoted the dissociation of PKEGs and increased the ionic layer of the slipping plane and the thickness of the double electric layer (36), resulting in a higher zeta potential. Similarly, an alkali pH-shift increased the zeta potential in a sunflower protein-based emulsion (14).

#### 3.6.5 Centrifugal stability

**Figure 6:**
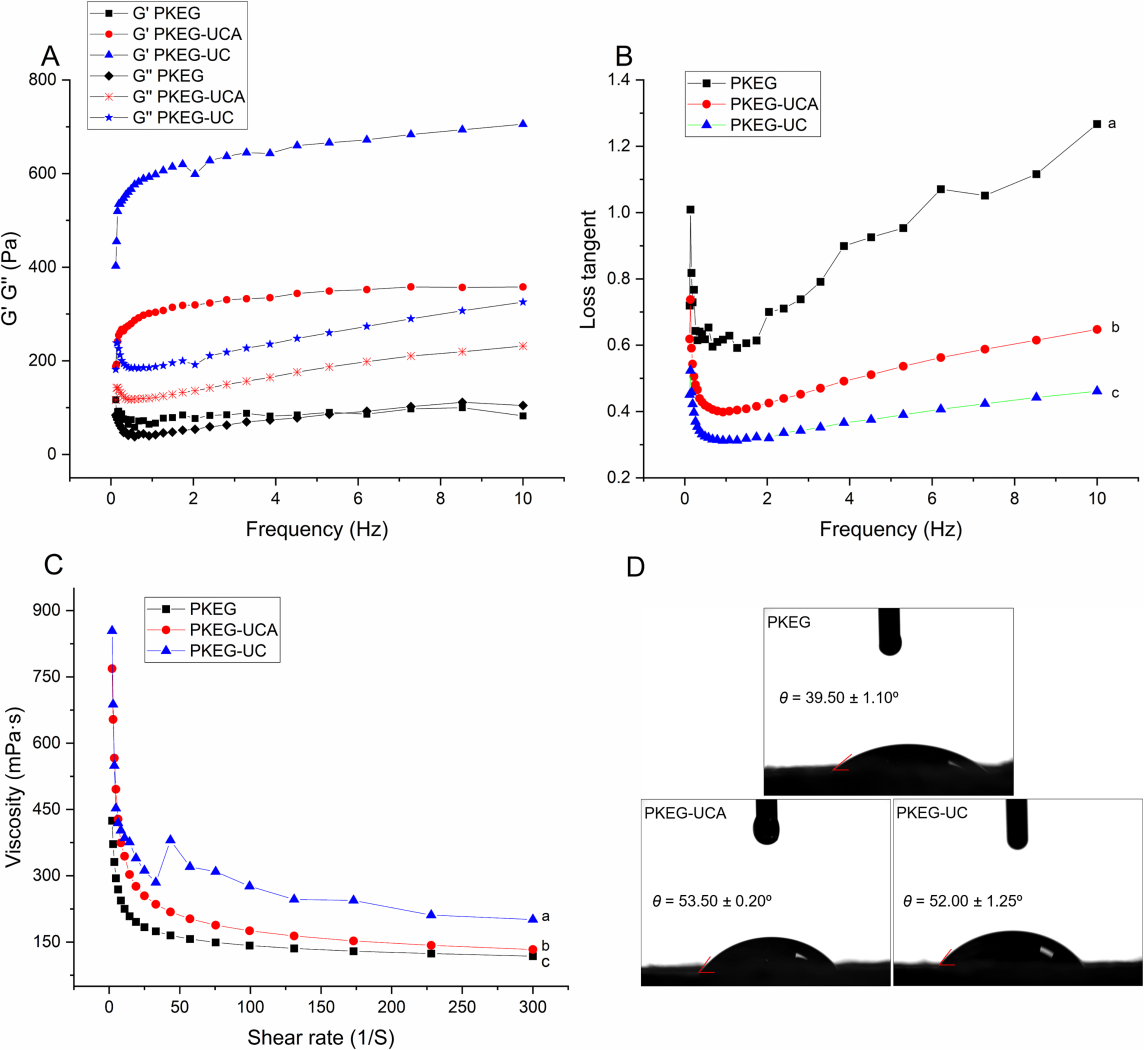
Rheological characteristics of the PKEG, PKEG-UCA, and PKEG-UC-based emulsions. **(A)** Energy storage modulus (*G*’) and loss modulus (*G*”), and **(B)** loss factor of the emulsions at various frequencies; **(C)** Viscosity at different shear rates; and contact angle **(D)**. Different lowercase letters (a–c) near the lines mean statistically significant difference (*p* < 0.05).

As depicted in Figure 5D, the PKEG-based emulsion showed lower centrifugal stability (36.59–45.52%) than that of the soy protein-based emulsion [54.91%; Krstonošić et al. (8)]. However, the PKEG-UC and PKEG-UCA-based emulsions showed better centrifugal stability at pH 2–10 (*p* < 0.05), which was ascribed to their higher random coil content (Figure 2B) and interface adsorption capacity (Figure 5A), lower loss tangent, and higher viscosity in the emulsions (Figures 6B,C). Greater structure flexibility and interface adsorption capacity are conducive to forming a stable emulsion film against centrifugal force (29). The higher viscosity and lower loss tangent of the modified PKEG emulsions confirmed the formation of a stable emulsion film, which is crucial for emulsion stability (35). Additionally, carboxymethylation and chlorogenic acid-grafting reduced droplet size and increased zeta potential (Figures 5B,C), thereby preventing droplet aggregation and enhancing centrifugal stability. Furthermore, the PKEG-UC-based emulsion exhibited better centrifugal stability than PKEG-UCA at pH 2, 8, and 10 (*p* < 0.05), indicating that carboxymethylation was more effective in enhancing the centrifugal stability.

The centrifugal stability of the PKEG, PKEG-UC, and PKEG-UCA-based emulsions was lowest at pH 4.0, attributed to the lowest electrostatic repulsion and physical barrier forces between droplets under this condition (36). The centrifugal stability of PKEG-UC-based emulsion increased as the pH shifted from 6 to 10, attributed to increased zeta potential, decreased droplet size, and reduced loss tangent under an alkali pH-shift (Figures 5B,C, 6B), ultimately facilitating the formation of a stable network-like emulsion film. Similarly, prior studies have shown that carboxymethylation and chlorogenic acid binding enhanced the centrifugal stability of the spent grain and albumin-stabilized emulsions (17, 19).

#### 3.6.6 Rheological properties

A lower loss tangent (tan*δ* = *G”/G’*) indicates greater emulsion stability, whereas a high loss tangent (> 1) suggests pronounced viscous dominance and correspondingly reduced structural integrity (8). As shown in Figure 6A, the PKEG-based emulsion exhibited *G”* > G’ at a frequency above 6 Hz, corroborating its elevated tan δ (> 1) (Figure 6B) and confirming its relatively poor colloidal stability (Figure 3D). Conversely, both the PKEG-UC- and PKEG-UCA-stabilized emulsions maintained tan δ < 1 across the measured frequency range, reflecting their enhanced interfacial rigidity and the formation of a cohesive, gel-like interfacial network (12), which would significantly improve emulsion stability. Therefore, ultrasonication-assisted chlorogenic acid grafting or carboxymethylation markedly increased the interfacial adsorption capacity of PKEG (Figure 5A), concurrently reducing droplet size and elevating the zeta potential (Figures 5B,C). These effects were conducive to the formation of a robust, viscoelastic interfacial film and a concomitant reduction in loss tangent (29).

The PKEG, PKEG-UC, and PKEG-UCA-based emulsions exhibited characteristic shear-thinning behavior (Figure 6C), confirming their classification as non-Newtonian fluids (35). Notably, the PKEG-UC and PKEG-UCA-stabilized emulsions displayed higher apparent viscosity than the PKEG-based emulsion, indicating that the chemical modification augmented interfacial film viscosity and mechanical resilience. Among them, the PKEG-UCA-based emulsion achieved the lowest loss tangent and highest viscosity (Figure 3D), correlating with its superior emulsion stability. Xing et al. (29) demonstrated that phenolic acid- conjugation improved the stability of egg white protein-based emulsions primarily through viscosity-mediated reinforcement of the interfacial layer.

#### 3.6.7 Contact angle

Contact angle is a key indicator that reflects emulsion type and the ability of emulsifiers to wet the oil and aqueous phases (10). As shown in Figure 6D, the contact angles of the PKEG-, PKEG-UC-, and PKEG-UCA-stabilized emulsions (39.5°–53.50°) were all below 90°, indicating that these emulsions belong to the oil-in-water type (O/W) (35). The PKEG-UC and PKEG-UCA-stabilized emulsions displayed bigger contact angles (52.00° and 53.50°, respectively) than that of the PKEG-based emulsion. Since emulsion stability is positively correlated with the contact angle approaching 90° (35), the larger contact angles of PKEG-UC- and PKEG-UCA-stabilized emulsions contributed to their superior stability, which was consistent with the results presented in Figure 3D. After ultrasonication-assisted carboxymethylation and chlorogenic acid grafting, the hydrophilicity and hydrophobicity of PKEG were improved (Figures 3A,B), respectively, and its structural flexibility was increased (Figures 1, 2), enhancing its wettability in the aqueous and oil phases in emulsions, thereby increasing the contact angle (8).

Although this study revealed some clear mechanisms, including effects of the modifications on the molecular mass, random coil content, hydrophobicity of PKEG, and the zeta potential, particle size, rheological properties, and contact angle in the emulsion, more direct interfacial evidence is needed for further elucidation and optimization of emulsification properties, such as investigations of dynamic interfacial tension, adsorption kinetics, interfacial rheology/film strength, and competitive displacement.

## 4 Conclusion

This study demonstrated that carboxymethylation, chlorogenic acid grafting, and ultrasonication-assisted chlorogenic acid grafting or carboxymethylation altered the subunit composition and secondary structure of PKEG. Ultrasonication-assisted carboxymethylation proved to be most effective in enhancing the emulsifying activity (57.06–109.42 m^2^/g) and emulsion stability (64.72–90.00%) of PKEG via reducing its molecular mass; increasing its random coil content (25.9–52.4%), solubility (59.14–86.85 g/100 g), and interface adsorption capacity (58.23–245.61 μg/mL); decreasing the droplet size (1.24–0.69 μm) and loss tangent; and enhancing the zeta potential (−33.07 to −76.54 mV), centrifugal stability (26.59–74.14%), and bulk contacting angle and viscosity. Moreover, ultrasonication-assisted chlorogenic acid grafting increased both the random coil and *β*-sheet contents, resulting in an increase in surface hydrophobicity (from 276.49–351.38), thereby enhancing the emulsifying activity (57.06–82.54 m^2^/g) and emulsion stability (64.72–89.96%) of PKEG. Importantly, chlorogenic acid-grafting mitigated the sensitivity of PKEG’s solubility and emulsifying performance to pH variation. Ultrasonication enhanced the improvement effect of carboxymethylation and chlorogenic acid grafting on the emulsifying properties of PKEG. Furthermore, an alkaline pH shift enhanced the solubility and emulsifying properties of the tested PKEGs. Palm kernel expeller is an abundant and low-cost protein resource. The findings of this study indicated that PKEG-UC and PKEG-UGC can be used as low-cost and efficient emulsifiers and emulsion stabilizers. Nevertheless, confirming the safety of PKEG-UCA and its byproducts requires further investigation, and further studies are needed to explore the specific mechanisms of these modifications. Finally, the effects and mechanisms of single ultrasonication, carboxymethylation, or chlorogenic acid grafting on the emulsifying properties of PKEG require further investigation.

## Data Availability

The original contributions presented in the study are included in the article/supplementary material, further inquiries can be directed to the corresponding author.
